# The Liver–Eye Axis of Dietary Vitamin A Homeostasis: A Review of Mechanisms, Receptors, and Visual Outcomes

**DOI:** 10.3390/nu18050803

**Published:** 2026-02-28

**Authors:** Sophie Gao, Matthias Leung, Rakesh Radhakrishnan, Glenn Prazere Lobo

**Affiliations:** Department of Ophthalmology and Visual Neurosciences, University of Minnesota, Lions Research Building, 2001 6th Street SE, Minneapolis, MN 55455, USAleung132@umn.edu (M.L.); rakeshr@umn.edu (R.R.)

**Keywords:** retinol-binding protein 4, retinol-binding protein 4 receptor 2, all-*trans*-retinol, stimulated by retinoic acid 6 receptor, retinoids, liver–eye axis

## Abstract

**Background:** Vitamin A is an essential micronutrient critical for vision, immune function, cellular differentiation, and metabolic homeostasis. The liver serves as the primary site of vitamin A storage and systemic distribution, delivering all-*trans*-retinol (ROL) to peripheral tissues, including the retina, via retinol-binding protein 4 (RBP4). Tight regulation of retinoid delivery to peripheral tissues is crucial for metabolic function and photoreceptor integrity. **Objectives:** This review provides a current understanding of intestinal absorption, hepatic storage, systemic transport, and ocular utilization of vitamin A, with a focus on the role of retinol-binding protein 4 receptor 2 (RBPR2) in mediating liver–eye communication. **Results:** Studies using *Rbpr2* knockout mice show that loss of RBPR2 impairs hepatic ROL-bound RBP4 uptake and retinyl ester concentrations, alters circulating holo-RBP4 levels, and reduces ocular retinoid content, leading to visual dysfunction and photoreceptor structural abnormalities. These effects are amplified under dietary vitamin A-deficient conditions, highlighting its unique sensitivity to tightly regulated serum RBP4-ROL transport. In mouse models of Stargardt disease, dietary modulation of RBPR2 mRNA expression and serum RBP4-ROL levels protects against lipofuscin accumulation and attenuates retinal cell degeneration, suggesting translational relevance. **Conclusions:** This review article explores the liver–eye axis by focusing on the regulation of retinoid homeostasis in the liver and other systemic organs through the non-ocular RBP4 receptor protein, RBPR2, and how RBPR2 expression may influence liver and serum retinoid homeostasis, which can impact visual function. Disruption of RBPR2 markedly compromises systemic and retinal retinoid supply, emphasizing its potential as a therapeutic target for metabolic and retinal disorders.

## 1. Introduction

In this review, we first outline the pathway of all-*trans*-retinol from intestinal absorption to hepatic storage and its subsequent mobilization to the eye, where it is essential for visual function. We then summarize emerging evidence supporting a central role for the liver in systemic retinoid homeostasis, with particular emphasis on recent studies of the retinol-binding protein 4 receptor 2 (RBPR2), a receptor predominantly expressed in hepatic tissue. Finally, we discuss the current understanding of RBPR2 in whole-body retinoid transport and regulation and consider how the modulation of RBPR2 activity may provide therapeutic opportunities for retinal degenerative disorders, including Stargardt disease.

### 1.1. Retinol Intake and Storage in the Liver

**Figure 1:**
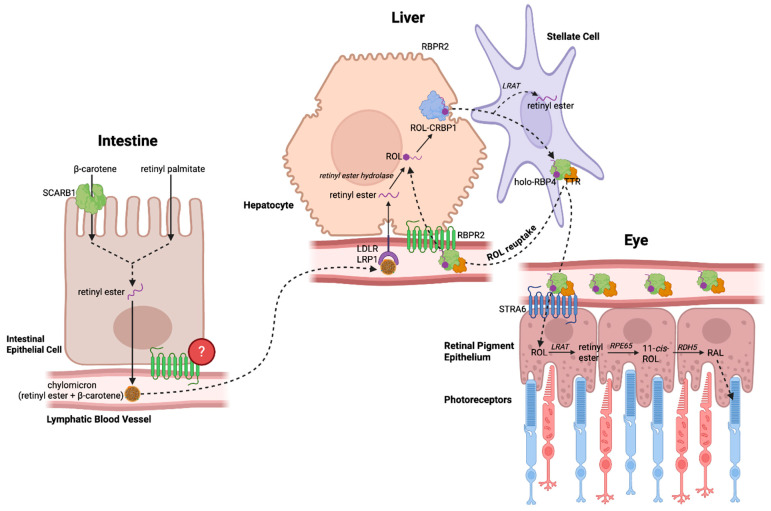
Overview of dietary vitamin A metabolism and transport along the liver–eye axis. Dietary vitamin A is absorbed in the intestine and trafficked to the liver as chylomicron-associated retinyl esters, establishing the liver as the central hub for vitamin A storage and mobilization [1,2,3,4,5,6,7]. Hepatic processing generates circulating all-*trans*-retinol (ROL), which is distributed systemically as an RBP4-TTR-ROL complex. Delivery of ROL to the eye supports retinal uptake via STRA6 and sustains the visual cycle within photoreceptors [8,9]. Within this liver–eye axis, RBPR2 emerges as a key regulator of retinoid flux by controlling hepatic reuptake of circulating ROL and potentially modulating intestinal ROL transport, thereby coordinating systemic retinoid homeostasis with visual demand [4,10,11]. Abbreviations: SCARB1, scavenger receptor class B type 1; LDLR, LDL receptor; LRP1, LDL receptor-related protein 1; RBPR2, retinol-binding protein 4 receptor 2; ROL, all-*trans*-retinol; CRBP1, cellular retinol-binding protein 1; LRAT, lecithin retinol acyltransferase; RPE65, retinol isomerohydrolase or retinal pigmented epithelium 65 kDa protein; RDH5, retinol dehydrogenase 5; STRA6, stimulated by retinoic acid 6; TTR, transthyretin; RBP4, retinol-binding protein 4. Created with BioRender.com.

Before dietary vitamin A is stored in the liver, it must be absorbed through the intestine and distributed in the circulation via chylomicrons. There are two pathways through which dietary vitamin A is obtained: provitamin A carotenoids (β-carotene) from plant sources and pre-formed vitamin A (retinyl palmitate) from animal sources. These pathways converge when both forms of vitamin A are converted into retinyl esters (RE), mainly retinyl palmitate, which are then packaged into chylomicrons and released into systemic circulation through the lymphatic system (Figure 1) [1,2].

The liver is the main site of storage for vitamin A, accounting for approximately 70% of total retinoids in the body [3]. Within the liver, hepatocyte-associated receptor proteins, including LDL-receptor (LDLR), the LDL-receptor-related protein 1 (LRP1), SR-B1 and heparan sulphate proteoglycans (HSPGs), mediate uptake of circulating chylomicrons [2]. Once inside the hepatocytes, retinyl esters are hydrolyzed by retinyl ester hydrolases to form retinol, which binds the cellular retinol-binding protein, type I (CRBPI) [2,4]. The retinol–CRBP1 complex is then either transported to hepatic stellate cells, where it is re-esterified by lecithin retinol acyltransferase (LRAT) and stored as retinyl esters, or transferred to the retinol-binding protein (RBP4) for release into systemic circulation (Figure 1) [1,2,4].

### 1.2. All-Trans-Retinol Transport Is Mediated by RBP4 Protein

Retinoids exist in systemic circulation as all-*trans*-retinol (ROL) bound to retinol-binding protein 4 (RBP4). First discovered in 1968 by Kanai et al., RBP4 was purified from human plasma and found to bind retinol in a 1:1 ratio [5]. In addition, it was discovered that retinol bound to RBP4 circulates in a complex with prealbumin, also known as transthyretin (TTR) [1,5]. Based on studies on TTR-deficient mice, it has been suggested that TTR enhances, but is not required for, the secretion of RBP4 [6,12,13]. The concentration of RBP4 in the blood is highly regulated even upon variable dietary uptake of retinoids, remaining within a range of 2–3 μM in humans and 1 μM in mice [1,14]. Altered circulating RBP4 levels have been implicated in several disease states, notably in insulin resistance and cardiovascular disease [15]. Yang et al. showed that serum RBP4 levels are elevated in adipose-specific *Glut4*^−/−^ mice, a model of insulin resistance, while treatment with an insulin-sensitizing drug normalizes RBP4 levels [16]. In human patients, elevated serum RBP4 levels correlated with the magnitude of insulin resistance, as well as other cardiovascular risk factors linked to type 2 diabetes [17]. Furthermore, elevated serum RBP4 levels have been reported in patients with clinical arteriosclerosis, high-grade carotid stenosis, inflammatory dilated cardiomyopathy, coronary artery disease, and advanced heart failure [15,18,19,20,21,22,23].

Studies have also shown the effects of RBP4 expression on visual function. The work of Quadro et al. showed that loss of RBP4 in mice combined with a vitamin A-deficient diet resulted primarily in visual defects, underscoring the importance of RBP4 in ROL transport to the eye, especially in comparison to other peripheral organs. Interestingly, the same study found that mice lacking RBP4 but maintained on a normal chow diet exhibit impaired vision only during the first few months of life, with visual function normalizing by 4–5 months of age [7]. A more recent study, however, used RBP4-deficient mice generated on a C57BL/6 genetic background and found that visual defects were pronounced even at 40 weeks of age, despite a vitamin A-sufficient diet [24]. Overall, these studies suggest that although alternative, less efficient pathways for ROL delivery to the eye may exist, RBP4 remains an essential mediator of ROL mobilization from the liver required for normal visual function.

### 1.3. Serum RBP4 Receptors: STRA6 and RBPR2

**Figure 2:**
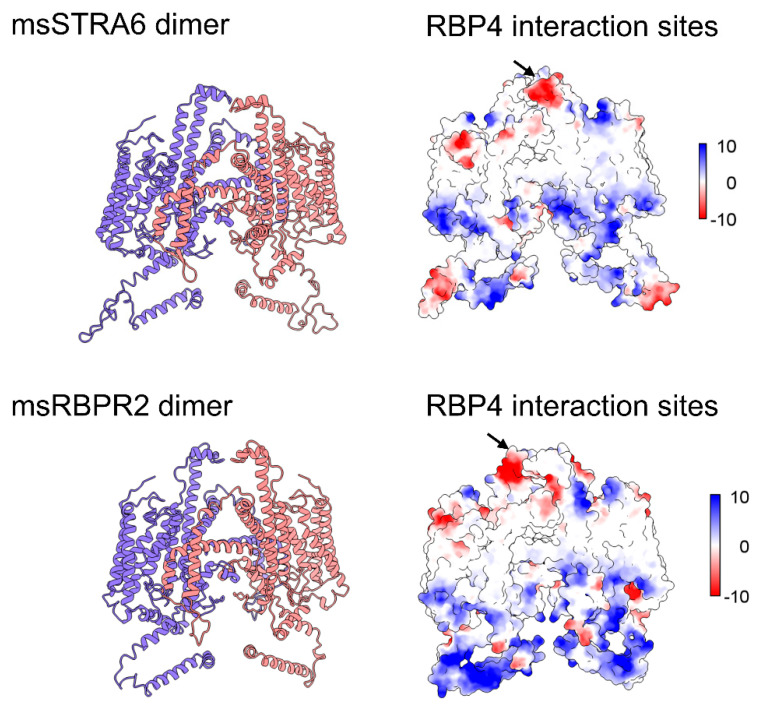
Protein structure homology modelling of mouse STRA6 and RBPR2 protein. STRA6 dimer structure generated using template PDB-5SY1, showing electrostatic surface and extracellular RBP4 binding domain (arrows) using UCSF ChimeraX analysis software. Previous wet lab and in silico docking studies showed protein–protein interactions between the RBP4-RBPR2 dimer and retinol interaction sites in RBPR2, indicating possible binding and internalization regions for retinol from the extracellular matrix to the cytosol.

For many years, the mechanism by which circulating ROL bound to retinol-binding protein 4 (holo-RBP4) is taken up by peripheral tissues remained poorly understood. It was not until 2007 that Kawaguchi et al. first identified the high-affinity RBP4 membrane receptor, stimulated by retinoic acid 6 (STRA6), in bovine retinal pigment epithelium (RPE) cells [8]. ROL-bound RBP4 in the bloodstream is taken up into the retinal pigment epithelium (RPE) through facilitated transport by STRA6. There, ROL is converted to RE by LRAT and then isomerized by RPE65 to 11-*cis*-ROL, where it enters the visual cycle. *STRA6* mutations in humans have been implicated in Matthew–Wood syndrome, a disorder characterized by bilateral anophthalmia along with syndromic defects, including pulmonary hypoplasia, diaphragmatic anomalies, congenital heart defects, and intellectual disability [1,25]. Similar features have also been observed in STRA6-deficient zebrafish, with low retinoid levels in the eye resulting in ocular abnormalities, in addition to cardiac and craniofacial malformations [9]. Although STRA6 is expressed in several systemic organs and tissues, including the spleen, kidneys, and lungs, it is absent in the liver, the primary site of retinoid storage [4]. This prompted the question: is there another RBP4 receptor that mediates the reuptake of circulating holo-RBP4 into the liver? In 2013, the Graham Lab made a significant advancement toward answering this knowledge gap with the discovery of a novel high-affinity RBP4 receptor structurally related to that of STRA6 (Figure 2) [26]. This receptor, named retinol-binding protein 2 receptor-2 (RBPR2) or STRA6like (STRA6l), localized to the plasma membrane of cultured hepatocytes and increased ROL uptake upon incubation with exogenous holo-RBP4, possibly enhanced through interactions with LRAT. In zebrafish, RBPR2 expression has been identified in the liver and intestine, as well as in the pancreas to a lesser extent [27]. In wild-type mice, mRNA analysis further confirmed the presence of RBPR2 expression in the liver and kidneys, as well as in the intestine, suggesting a role in dietary retinol absorption. It was also found to be upregulated during adipocyte differentiation [26]. While STRA6 has been shown to mediate bidirectional shuttling of retinol, the bidirectionality of retinol shuttling by RBPR2 remains unclear. Studies have validated RBPR2-mediated retinol influx in hepatocytes; however, the capability of RBPR2 to mediate retinol efflux remains hypothetical, requiring further investigation (Figure 1 and Table 1) [26,28].

### 1.4. Retinoids in the Visual Cycle

Circulating holo-RBP4 eventually reaches the eye, where it is involved in the visual cycle. As previously mentioned, the holo-RBP4 complex interacts with the cell surface receptor STRA6 in the RPE cells, which internalizes ROL. There, ROL is esterified by LRAT to form all-*trans*-retinyl esters, which are then isomerized by retinoid isomerohydrolase (RPE65) into 11-*cis*-retinol and subsequently oxidized to 11-*cis*-retinal (RAL) by the retinol dehydrogenase RDH5 [29]. RAL is then transported from the RPE to the photoreceptors, where it acts as the chromophore attached to an opsin G protein-coupled receptor (GPCR). Upon light activation by a photon, RAL undergoes photoisomerization into all-*trans*-retinal (atRAL), inducing a conformational change in the opsin GPCR that leads to a signal cascade and hyperpolarizes the photoreceptor cell [30].

Thus, to maintain photosensitivity of photoreceptor cells, RAL is regenerated through a series of enzymatic reactions. First, atRAL is released from the opsin following the photoisomerization reaction and is reduced to ROL by the photoreceptor-specific retinol dehydrogenase (RDH8) [29,30,31]. ROL then localizes back to the RPE and undergoes esterification by LRAT to become an RE, which is further converted to back 11-*cis*-retinol by RPE65 [29,32,33,34]. Finally, 11-*cis*-retinol is oxidized back to RAL and recombines with an opsin, thereby continuing the visual cycle [29,30].

### 1.5. Whole-Body Retinoid Homeostasis Begins in the Liver

The liver is an organ that is involved in numerous essential metabolic pathways, including glucose, amino acid, lipid, vitamin, and mineral metabolism, as well as the detoxification of ammonia and bilirubin and the production of immune factors [35]. Consequently, hepatic dysfunction has significant effects on the functioning of extra-hepatic tissues, including that of the eye [35,36]. The connections between liver and eye function have been increasingly investigated, as the liver produces numerous secretory factors and signaling proteins that influence ocular processes. These include maintaining the structural integrity of the ocular components and the regulation of inflammation and angiogenesis [36]. Liver dysfunction has been associated with several ocular pathologies. For example, multiple studies have demonstrated an association between the severity of nonalcoholic fatty liver disease (NAFLD) and increased intraocular pressure and glaucoma risk [35,37,38,39]. Other ocular diseases, such as age-related macular degeneration, type II diabetes mellitus-associated retinal dysfunction, dry eye disease, and ocular motor cranial nerve palsies, have also been linked to liver-related risk factors [35]. However, studies specifically examining liver–eye crosstalk mediated by vitamin A receptors and retinoids remain limited.

### 1.6. The Role of RBPR2 in Liver Retinoid Homeostasis and Its Effects on Vision

**Figure 3:**
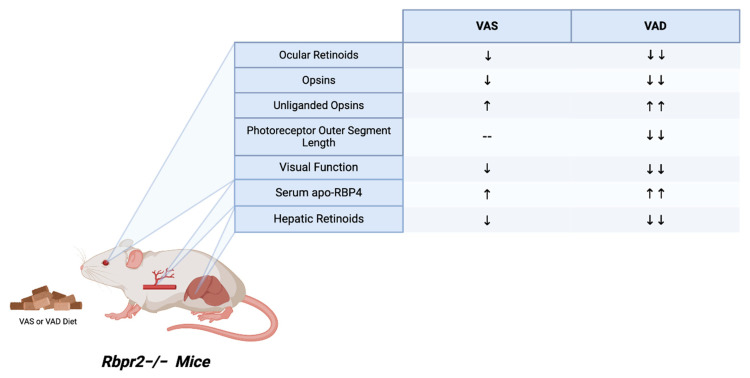
Multi-organ phenotypes in *Rbpr2*^−/−^ mice at 6-months of age. *Rbpr2*-knockout (*Rbpr2*^−/−^) mice maintained on vitamin A-sufficient (VAS) or vitamin A-deficient (VAD) diets display alterations in ocular, hepatic, and serum retinoid homeostasis, accompanied by reduced visual function compared with wild-type controls [10,11]. Retinoid concentrations were decreased in both ocular and hepatic tissues, while serum levels of unliganded RBP4 protein (apo-RBP4) were elevated. These effects were more pronounced under VAD conditions, which additionally led to significant shortening of photoreceptor outer segments [11]. RBP4, serum retinol-binding protein 4; VAS, vitamin A-sufficient diet; VAD, vitamin A-deficient diet. Downward arrows, decreased expression/concentrations/function; Upward arrows, high expression/concentrations/function; hypen, no significant photoreceptor phenotype. Created with BioRender.com.

Recently, our lab further elucidated the role of the second RBP4-ROL receptor, RBPR2, in retinoid homeostasis through studies with *Rbpr2*-knockout (*Rbpr2*^−/−^) mice fed either vitamin A-sufficient (VAS) or vitamin A-deficient (VAD) diets. High-performance liquid chromatography (HPLC) analysis of serum collected at the 6-month time point revealed impaired serum holo-RBP4 complex homeostasis (Figure 3). Specifically, *Rbpr2*^−/−^ mice demonstrated higher levels of the RBP4 protein in the serum compared to the wild-type mice. Second, serum ROL levels were decreased in *Rbpr2*^−/−^ mice compared to wild-type mice, indicating a higher proportion of unliganded serum apo-RBP4 protein in *Rbpr2*^−/−^ mice. Furthermore, significant differences in serum RBP4 protein and ROL levels were observed between *Rbpr2*^−/−^ mice fed under VAS or VAD conditions. Together, these findings demonstrate that mice lacking the RBPR2 receptor were more susceptible to disruptions in serum holo-RBP4 homeostasis under conditions of varying dietary vitamin A intake (Figure 3) [11].

The disruption in serum holo-RBP4 homeostasis further extends to disruptions in the eye. Under both vitamin A dietary conditions, but more markedly with the VAD diet, *Rbpr2*^−/−^ knockout mice exhibited pathological changes in the ocular tissue and a decrease in visual function. Upon HPLC and spectrophotometry examination, the ocular tissues contained decreased ocular retinoid levels and opsins, respectively. Furthermore, *Rbpr2*^−/−^ knockout mice fed the VAD diet, but not the VAS diet, also exhibited shorter photoreceptor outer segments, suggesting that a diet high in vitamin A is able to partially rescue this phenotype. Interestingly, while there was a decrease in total retinoid concentration in the liver and other peripheral tissues, the pathology in these non-ocular tissues was unremarkable. This suggests that retinoid levels in non-ocular systemic organs may be maintained to a greater extent by circulating retinyl esters in chylomicrons. Overall, the *Rbpr2*^−/−^ knockout mouse demonstrated that the disruption of retinol hemostasis and storage in the liver mediated by the RBPR2 receptor has profound effects on ocular pathology (Figure 3) [10].

### 1.7. Modulation of RBPR2 in a Mouse Model of Stargardt Disease

**Figure 4:**
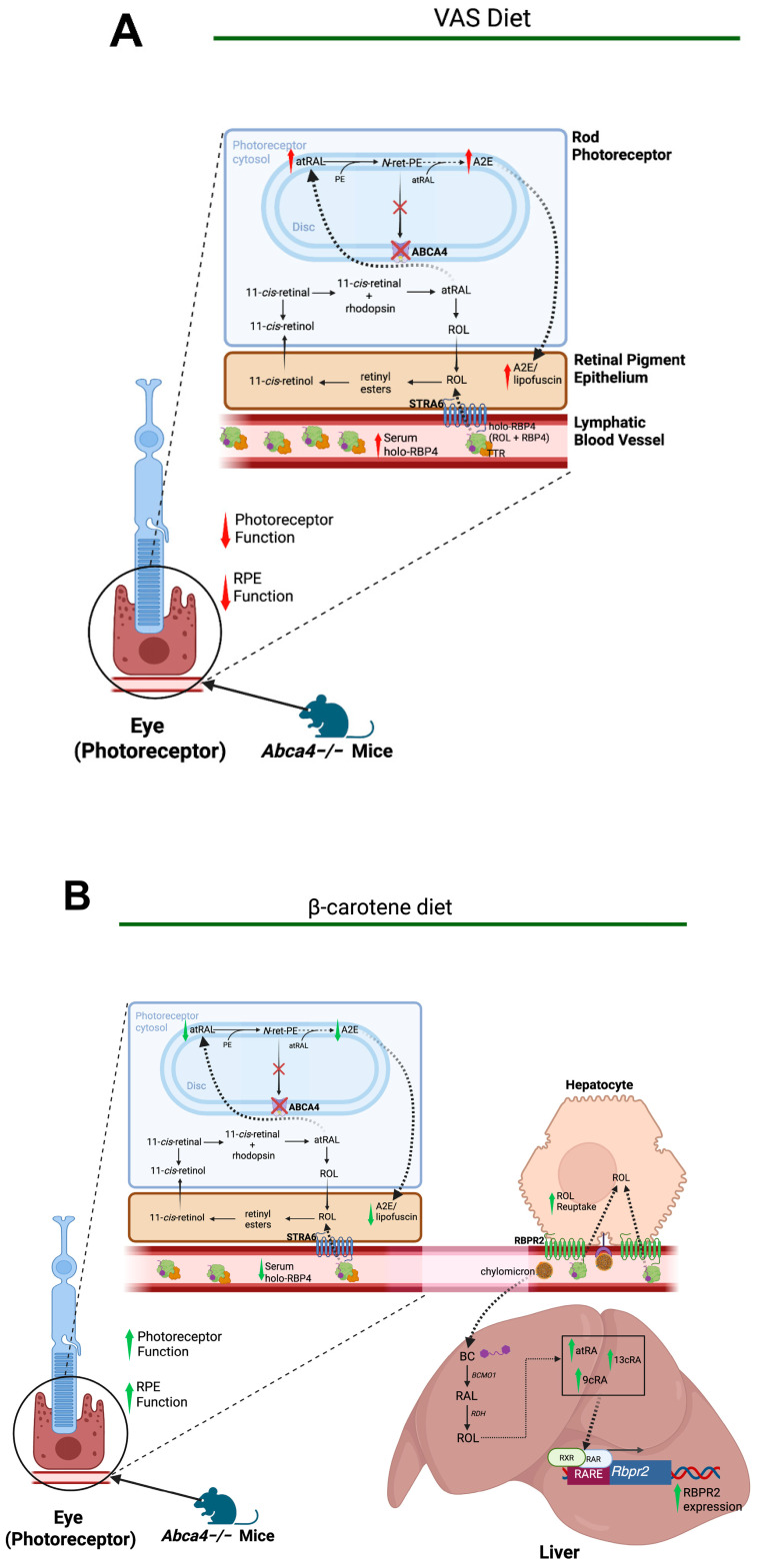
Effects of β-carotene (BC)-supplemented diets on lipofuscin accumulation and visual function in the *Abca4*^−/−^ mouse model of Stargardt disease. In the visual cycle, *N*-retinylidene-phosphatidylethanolamine (*N*-ret-PE), a byproduct of all-*trans*-retinal (atRAL) condensation with phosphatidylethanolamine (PE), can react further with atRAL to form A2E, a major component of lipofuscin [44,45]. (**A**) Loss of the ABCA4 flippase, which normally transports *N*-ret-PE to the cytoplasmic side of photoreceptor discs, results in A2E accumulation within the retinal pigment epithelium (RPE). This phenotype persists in an *Abca4*^−/−^ mouse fed a VAS diet [11]. (**B**) *Abca4*^−/−^ mice fed a BC-supplemented diet exhibited increased hepatic production of retinoic acid isomers (atRA, 13cRA, and 9cRA) that bind RAR and RXR elements within the *Rbpr2* promoter, upregulating *Rbpr2* mRNA expression. Enhanced RBPR2 activity in hepatocytes promoted retinol (ROL) reuptake and storage, reducing circulating holo-RBP4 concentrations [11]. Consequently, decreased systemic holo-RBP4 levels attenuated ocular A2E/lipofuscin accumulation, leading to improved photoreceptor and RPE function. In contrast, mice maintained on a VAS diet lacked this protective effect. Abbreviations: A2E; *N*-retinylidene-*N*-retinylethanolamine; ROL; all-*trans*-retinol; ABCA4; ATP-binding cassette, subfamily A; RARE, retinoic acid response element; RXR, retinoid X receptor; RAR, retinoic acid receptor; BC, β-carotene; RDH, retinol dehydrogenase; BCMO1; β-carotene mono oxygenase 1; STRA6, stimulated by retinoic acid 6; RBPR2, retinol-binding protein 4 receptor 2. Created with BioRender.com.

The liver–eye axis mediated by RBPR2 may also be useful in studying Stargardt disease (STGD1). STGD1 is the most common inherited form of macular degeneration, with an estimated incidence of 1:10,000. It is characterized by juvenile or young adult onset of progressive bilateral atrophy of the foveal retinal pigment epithelium (RPE) and deposition of orange-yellow flecks of lipofuscin-like material around the macula and/or mid-retinal periphery, leading to impaired central vision [40,41,42]. There is currently no approved treatment to date. Variants in the *ABCA4* gene have been shown to cause STGD1 [42]. This gene encodes the ABCA4 protein, an ATP-binding cassette transporter responsible for translocating *N*-retinylidene-phosphatidylethanolamine (*N*-ret-PE) from inside the disks to the cytoplasmic side of the disk membrane, preventing its accumulation in the RPE [43]. *N*-ret-PE is a precursor of several toxic retinoid compounds, including *N*-retinylidene-*N*-retinylethanolamine (A2E), the major fluorophore of lipofuscin (Figure 4) [44].

Given this etiology, the modulation of serum RBP4-ROL availability, and thereby ROL availability to the eye, has been investigated as a method to reduce the accumulation of lipofuscin bisretinoids in the RPE [45]. Several RBP4 antagonists that prevent the binding of retinol to RBP4 have been shown to successfully reduce serum RBP4-ROL levels [45,46,47,48,49,50]. One of these compounds, the non-retinoid RBP4 antagonist A1120, decreased serum RBP4 levels by 75% and lipofuscin bisretinoid levels by 50% in *Abca4*^−/−^ mice [48]. Currently, there are two RBP4 antagonists, tinlarebant and STG-001, undergoing clinical trials for the treatment of STGD1 [45].

Building on the hypothesis that reducing circulating RBP4-ROL levels would attenuate toxic retinoid accumulation in the STGD1-phenotype, we investigated dietary interventions to modulate RBPR2 activity in the *Abca4*^−/−^ mouse model of STGD1. First, we identified multiple RAR and RXR sites on the murine *Rbpr2* gene promoter that were inducible by exogenous and dietary β-carotene (BC) metabolites. Next, we showed that feeding *Abca4*^−/−^ mice a BC-supplemented diet induced Rbpr2 expression, which subsequently decreased serum RBP4 levels, reduced ocular A2E accumulation, and improved photoreceptor and RPE function. Conversely, *Abca4*^−/−^ and *Rbpr2*^−/−^; *Abca4*^−/−^ mice fed a BC-deficient diet did not show this protective effect (Figure 4A vs. Figure 4B) [51]. Together, these findings highlight the potential of β-carotene-supplemented diets as a non-pharmacologic intervention to attenuate the progression of Stargardt disease through the reduction in serum RBP4.

### 1.8. Clinical Applications and Safety Considerations of β-Carotene Supplementation

However, certain health considerations exist regarding the exogenous consumption of β-carotene. Of particular significance, β-carotene supplementation was shown to be associated with higher occurrences of lung cancer in subjects with a prior history of cigarette smoking. One of the most prominent studies conducted in support of this observation was the Beta-Carotene and Retinol Efficacy Trial (CARET) conducted in 1988, which investigated the effects of β-carotene and retinyl palmitate supplementation on incidences of cancer, heart disease, and total mortality within study subjects with occupational exposure to asbestos and subjects with a history of cigarette smoking. CARET was stopped 21 months early due to the clear evidence of harm towards its subjects, with 28% increased cases of lung cancer and 17% increased cases of mortality [52]. Numerous other studies have since supported the adverse results of β-carotene supplementation for populations with a prior history of cigarette smoking, as seen in CARET [53,54,55,56]. It should be mentioned, however, that this association was not seen in non-smokers or former smokers, where β-carotene was shown to have a protective effect [57].

Of clinical significance, the Age-Related Eye Diseases Study (AREDS), which sought to develop a supplement that reduces the risk of progression of age-related macular degeneration (AMD), removed the addition of β-carotene as a direct response to these studies, given the significant percentage of smokers within the general population. In the second iteration of the supplement (AREDS2), β-carotene was replaced with the non-provitamin A carotenoids lutein and zeaxanthin, and subsequent studies revealed that AREDS2 has shown no association with lung cancer for smokers while preserving its association with reduction in progression in AMD [58]. The exact mechanism for the association between smoking and β-carotene is not fully understood, but it may also be related to its involvement within retinoic acid signaling, the disruption of retinoid pathways as a whole, or its pro-oxidative effects within the oxygen-rich environment of the lung [57,59].

As such, the capability of β-carotene to modulate retinoic acid signaling in STGD1 is promising but warrants appropriate caution if administered as a supplement, and it may pose less concern when consumed as a natural part of the diet. As mentioned previously, the association between β-carotene and lung cancer was not observed for non-smokers and former smokers, and future clinical studies should restrict β-carotene treatments to only non-smoking populations. Moreover, alternate provitamin A carotenoids such as α-carotene or β-Cryptoxanthin have yet to be investigated deeply and could prove to circumvent the detractions seen in β-carotene.

### 1.9. Vitamin A Deficiency and Recommended Vitamin A Daily Intake

As previously described, *Rbpr2*^−/−^ mice are particularly susceptible to ocular phenotypes and vision loss when subjected to VAD conditions. These findings emphasize the importance of appropriate vitamin A transport and homeostasis in maintaining ocular health, particularly during periods of limited dietary intake. Here, we would like to further highlight the consequences of vitamin A deficiency in humans, which remains a major global public health concern. According to the World Health Organization (WHO), an estimated 250,000–500,000 children suffer from blindness as a direct result of vitamin A deficiency each year, posing a serious public health dilemma in its associated areas [60]. Vitamin A deficiency presents with a broad range of clinical manifestations. In vision, the earliest symptoms begin with nyctalopia, or night blindness. With progressing VAD, further degradation is characterized by the formation of Bitot’s spots within the conjunctiva. Under the conditions of continuing or severe VAD, a condition called xerophthalmia eventually develops, the leading cause of preventable childhood blindness. Xerophthalmia is characterized by corneal ulcers, scarring, and eventual blindness [61,62]. Beyond vision, other clinical manifestations of VAD include impaired taste [63,64], impaired weight gain [65], increased inflammation due to its role as an anti-inflammatory agent [66], and impaired reproduction and growth due to the role of vitamin A in various retinoid signaling pathways [67,68,69,70].

Currently, the Office of Dietary Supplements within the National Institutes of Health provides a table of recommended values for daily vitamin A intake (Table 2). Vitamin A intake recommendations are typically expressed as retinol activity equivalents. Provitamin A carotenoids contribute varying levels of RAE. β-carotene contributes 1/12 of RAE, α-carotene 1/24, and β-Cryptoxanthin 1/24. For preformed vitamin A, retinol has a one-to-one ratio contribution towards RAE, and one international unit (IU) of preformed vitamin A is equivalent to 0.3 micrograms of retinol [71,72]. Nutritionally, it is obtained either as preformed vitamin A (retinol and retinyl esters) from animal-derived foods or as provitamin A carotenoids (e.g., β-carotene) from plant sources. The recommended daily intake varies by age, sex, and physiological status, with adult requirements generally being around 800–900 µg retinol activity equivalents (RAE) per day, with increased needs during pregnancy and lactation (Table 2). Supplementation doses used in clinical or public health settings range from low-dose daily supplements to high-dose periodic regimens in deficiency-prone populations, particularly children. Major dietary sources of preformed vitamin A include liver, dairy products, eggs, and fortified foods, while provitamin A carotenoids are abundant in orange, yellow, and dark green vegetables such as carrots, sweet potatoes, spinach, and kale. Vitamin A deficiency remains a significant global health concern, especially in low- and middle-income countries, where it contributes to preventable blindness, increased susceptibility to infections, impaired growth, and higher morbidity and mortality in children. From a nutritional and public health perspective, adequate vitamin A intake is therefore critical, providing an important translational context for mechanistic insights derived from experimental and animal studies.

### 1.10. Limitations

Important interspecies differences exist in retinoid metabolism that should be considered when translating findings from animal models to human nutrition. For example, rodents efficiently convert dietary β-carotene to ROL due to high intestinal β-carotene 15,15′-monooxygenase (BCMO1) activity, whereas humans exhibit considerable interindividual variability in conversion efficiency, influenced by genetic polymorphisms and dietary fat intake. In addition, mice lack significant circulating β-carotene because of near-complete intestinal cleavage, in contrast to humans, in whom β-carotene is routinely detected in plasma and contributes directly to vitamin A status. Species differences in retinol transport further complicate translation; expression patterns and functional roles of retinol-binding protein receptors such as RBPR2 differ between rodents and humans, potentially affecting hepatic retinol uptake, mobilization, and tissue delivery. Moreover, commonly used laboratory diets for animals often contain preformed vitamin A at levels that exceed typical human intakes, bypassing intestinal carotenoid metabolism altogether. Together, these differences underscore the need for caution when extrapolating mechanistic insights from animal studies to human dietary recommendations and vitamin A supplementation strategies.

While this review synthesizes current knowledge on RBPR2 and retinoid biology, it also highlights several areas that warrant further investigation. A major priority is the extension of findings from preclinical models into human systems, as species-specific differences in retinoid metabolism and dietary vitamin A handling may limit direct translation. In particular, there is a clear need for functional studies examining RBPR2 expression, regulation, and activity in human tissues under physiological and disease-relevant conditions. Future research should also incorporate more rigorous control and reporting of dietary vitamin A status, including the form, dose, and duration of exposure, to reduce nutritional confounding and improve comparability across studies. In addition, expanding analytical frameworks beyond the RBPR2-RBP4 axis to encompass the broader retinoid signaling network, including alternative transporters, intracellular binding proteins, metabolic enzymes, and nuclear receptors, will be essential for capturing the full complexity of vitamin A homeostasis. Addressing these priorities through integrative nutritional, molecular, and clinical approaches will strengthen mechanistic interpretation and enhance the translational relevance of retinoid research.

## 2. Methods

This review article was based on a comprehensive survey of the peer-reviewed literature. Studies were identified through searches of PubMed and Google Scholar using combinations of keywords including *dietary vitamin A*, *retinoid homeostasis*, *RBP4*, *RBPR2*, *STRA6*, *liver–eye axis*, *retinal cell degeneration*, and *visual cycle*. Original research articles focusing on systemic retinoid metabolism and transport, as well as hepatic and ocular function, were included. Additional references were identified by reviewing the bibliographies of the relevant articles.

## 3. Conclusions

The coordinated transport of retinoids from the intestine to the liver and onward to the eye is essential for maintaining visual function. Throughout this pathway, multiple protein systems, including RBP4, STRA6, and RBPR2, all serve to facilitate retinol mobilization and cellular uptake. Evidence from *Rbpr2*-deficient mouse models has clarified the indispensable role of RBPR2 in sustaining systemic and ocular retinoid homeostasis, thereby establishing a mechanistic link between hepatic retinoid metabolism and retinal health. Collectively, these advances highlight the liver as a key regulatory hub of retinoid signaling and point to the modulation of hepatic retinol transport. Through dietary or pharmacological strategies, these mechanisms are promising therapeutic targets for retinal degenerative disorders characterized by retinoid toxicity, such as Stargardt disease.

### 3.1. Future Areas of Investigation

The non-ocular vitamin A receptor RBPR2 operates at the intersection of nutrient metabolism, systemic physiology, and visual health, positioning it as a key regulator of whole-body retinoid homeostasis. By coordinating the liver–eye axis, RBPR2 not only supports retinal integrity but may also influence systemic metabolic processes, including lipid metabolism, insulin sensitivity, and hepatic function. Elucidating these broader physiological roles has the potential to shift the conceptual framework of vitamin A biology from a predominantly retinal nutrient to a central mediator of metabolic and visual resilience. Such insights may ultimately inform new strategies for the prevention and treatment of retinal degeneration, metabolic disease, and their overlapping pathologies. Building on evidence that RBPR2 is essential for systemic retinoid homeostasis and retinal health, several priority areas for future investigation emerge.

First, it will be important to establish whether RBPR2 functions as a general mediator of retinal elasticity across diverse forms of stress and degeneration, including oxidative stress, lipid dysregulation, and age-related visual decline. Defining its role across distinct pathogenic contexts may identify RBPR2 as a unifying regulator of retinal integrity and a potential therapeutic target for age-related macular degeneration and other degenerative eye diseases.

Second, future studies should investigate the relationship between RBPR2-mediated retinoid transport and systemic metabolic health. Examining interactions between RBPR2, circulating RBP4, and pathways implicated in type 2 diabetes mellitus, metabolic dysfunction-associated steatotic liver disease, and nonalcoholic fatty liver disease may reveal previously unrecognized links between vitamin A biology and metabolic regulation.

Finally, identifying RBPR2-dependent molecular networks through integrated biochemical, transcriptomic, proteomic, and histological approaches will be critical. Such analyses may uncover molecular signatures that connect retinoid homeostasis with retinal and systemic metabolic function, enabling the discovery of novel biomarkers and therapeutic strategies at the interface of metabolism and visual health.

### 3.2. Integrative Perspective on RBPR2 in Retinoid and Metabolic Homeostasis for Visual Health

Collectively, this review article positions RBPR2 as a central integrator of retinoid transport, lipid metabolism, and retinal health, provides cross-disease validation of its function, and lays the foundation for therapeutic strategies targeting RBPR2 or apo-RBP4 signaling. By establishing RBPR2 as a nodal regulator connecting vitamin A biology with both ocular and systemic physiology, future studies have the potential to transform our understanding of nutrient-dependent mechanisms in health and disease and to guide interventions for retinal degeneration and metabolic disorders.

## Figures and Tables

**Table 1:** Comparative and distinct characteristics of the serum RBP4 receptors STRA6 and RBPR2. This table highlights the functional roles, directionality, and localization of the two RBP4-ROL receptors. While STRA6 is the primary receptor for ocular vitamin A uptake [4,8,9], RBPR2 facilitates systemic mobilization from the liver and intestine [4,10,27]. Mutations in *STRA6* have been implicated in various ocular pathologies [4,9,10,25,26,27].

	STRA6	RBPR2
**Primary Function**	Binds and transports ROL-bound RBP4.	
	Mediates tissue-specific retinol uptake specifically in the RPE.	Mediates liver retinol reuptake and regulates systemic retinoid bioavailability.
**Transport Direction**	Bidirectional (influx and efflux)	Influx; efflux capabilities are hypothesized but remain to be investigated.
**Tissue Expression (Mice)**	Eye (retinal pigment epithelium), brain, spleen, and lungs.	Liver, intestine, and adipose tissue.
	Kidneys	
**Tissue Expression (Zebrafish)**	Eye (retinal pigment epithelium), brain.	Liver, pancreas, intestine.
**Associated Pathologies**	Matthew–Wood syndrome (microphthalmia, anophthalmia, pulmonary hypoplasia, etc.) in humans; similar phenotype in STRA6-deficient zebrafish.	Implicated in metabolic phenotypes and ocular dysfunction in RBPR2-deficient mice and zebrafish; human disease associations have not yet been studied.

**Table 2:** Average daily recommended vitamin A intake listed in micrograms (mcg) of retinol activity equivalents (RAE) [60,61,71,72].

Life Stage	Recommended Amount
Birth to 6 months	400 mcg RAE
Infants 7–12 months	500 mcg RAE
Children 1–3 years	300 mcg RAE
Children 4–8 years	400 mcg RAE
Children 9–13 years	600 mcg RAE
Teen males 14–18 years	900 mcg RAE
Teen females 14–18 years	700 mcg RAE
Adult males	900 mcg RAE
Adult females	700 mcg RAE
Pregnant teens	750 mcg RAE
Pregnant women	770 mcg RAE
Breastfeeding teens	1200 mcg RAE
Breastfeeding women	1300 mcg RAE

## Data Availability

No new data were created or analyzed in this study. Data sharing is not applicable to this article.

## Abbreviations

RBPR2	retinol-binding protein 4 receptor 2
STRA6	stimulated by retinoic acid 6
ROL	all-*trans*-retinol
RA	all-*trans*-retinoic acid
RE	retinyl ester
RAL	11-*cis*-retinaldehyde
RPE	retinal pigmented epithelium
RBP4	retinol-binding protein 4
ABCA4	ATP-binding cassette, Subfamily A, Member 4
BC	β-carotene
